# Identification of a novel gene required for competitive growth at high temperature in the thermotolerant yeast *Kluyveromyces marxianus*

**DOI:** 10.1099/mic.0.001148

**Published:** 2022-03-25

**Authors:** Noemi Montini, Tyler W. Doughty, Iván Domenzain, Darren A. Fenton, Pavel V. Baranov, Ronan Harrington, Jens Nielsen, Verena Siewers, John P. Morrissey

**Affiliations:** ^1^​ School of Microbiology, APC Microbiome Ireland, Environmental Research Institute and SUSFERM Centre, University College Cork, Cork T12 K8AF, Ireland; ^2^​ Department of Biology and Biological Engineering, Chalmers University of Technology, SE-41296 Gothenburg, Sweden; ^3^​ School of Biochemistry and Cell Biology, University College Cork, Cork T12 K8AF, Ireland

**Keywords:** thermotolerance, non-conventional yeast, industrial biotechnology, genome annotation, ribosome profiling

## Abstract

It is important to understand the basis of thermotolerance in yeasts to broaden their application in industrial biotechnology. The capacity to run bioprocesses at temperatures above 40 °C is of great interest but this is beyond the growth range of most of the commonly used yeast species. In contrast, some industrial yeasts such as *Kluyveromyces marxianus* can grow at temperatures of 45 °C or higher. Such species are valuable for direct use in industrial biotechnology and as a vehicle to study the genetic and physiological basis of yeast thermotolerance. In previous work, we reported that evolutionarily young genes disproportionately changed expression when yeast were growing under stressful conditions and postulated that such genes could be important for long-term adaptation to stress. Here, we tested this hypothesis in *K. marxianus* by identifying and studying species-specific genes that showed increased expression during high-temperature growth. Twelve such genes were identified and 11 were successfully inactivated using CRISPR-mediated mutagenesis. One gene, *KLMX_70384*, is required for competitive growth at high temperature, supporting the hypothesis that evolutionary young genes could play roles in adaptation to harsh environments. *KLMX_70384* is predicted to encode an 83 aa peptide, and RNA sequencing and ribo-sequencing were used to confirm transcription and translation of the gene. The precise function of KLMX_70384 remains unknown but some features are suggestive of RNA-binding activity. The gene is located in what was previously considered an intergenic region of the genome, which lacks homologues in other yeasts or in databases. Overall, the data support the hypothesis that genes that arose *de novo* in *K. marxianus* after the speciation event that separated *K. marxianus* and *K. lactis* contribute to some of its unique traits.

## Introduction

A goal for a sustainable bioeconomy is to make the production of value-added chemicals independent of petroleum as starting material, in favour of sustainably sourced biomass [1]. Yeasts, used as microbial cell factories, represent a valuable tool in this goal, due to their ability to metabolize such biomasses, robust physiology, fermentation properties, genetic tractability and ability to tolerate harsh growth conditions [2]. Whilst *Saccharomyces cerevisiae* is the most widely used cell factory yeast, in recent years, attention has also focused on non-*Saccharomyces* species (‘non-conventional yeasts’) because of their diverse, industrially relevant traits [3]. As examples, due to its capacity to metabolize the sugar lactose as carbon source, *Kluyveromyces lactis* was proposed for lactose-rich dairy waste valorization [4]; the oleaginous yeast *Yarrowia lipolytica* can be exploited for lipid production [5]; thermotolerant yeasts such as *Kluyveromyces marxianus, Ogataea polymorpha* and *Ogataea thermomethanolica* can facilitate high-temperature processes such as simultaneous saccharification and fermentation [6, 7]; and finally, methylotrophic yeasts such as *Komagataella phaffii, O. polymorpha* and *O. thermomethanolica* have the capacity to successfully secrete high levels of proteins with post-translational modification such as glycosylation. The range of endogenous and heterologous promoters that are available in these yeasts make them very suitable for heterologous protein production [8–11]. A major barrier that limited opportunities to exploit the intrinsic advantageous properties of non-conventional yeasts for industrial processes is being overcome by the development of molecular tools for rapid strain engineering [2, 12–15].

Our particular focus here is on the food and industrial yeast *K. marxianus*. The broad substrate range conferred by a plethora of sugar transporters [16] and its thermotolerance [17] make this yeast an attractive host for biotechnological applications. Furthermore, the availability of genome editing and synthetic biology tools mean that there are few limitations to strain construction [13, 18, 19]. Nonetheless, further progress is required to improve knowledge of the physiological response of *K. marxianus* under industrially relevant stresses to allow wider use in biotechnological processes [20, 21]. One such response is thermotolerance, which is a desirable trait in strains used for industrial processes because it reduces cooling costs and allows fermentation at temperatures that mitigate bacterial contamination [22, 23]. *K. marxianus* is capable of growth at up to 45 °C, and sometimes higher [18]. It has therefore been considered for application in processes requiring high temperature, such as lignocellulosic biomass fermentation for bioethanol production [24–27]. Although *S. cerevisiae* is not intrinsically thermotolerant, responses and adaptation to high temperature have been quite widely studied in this model species. Among the known heat stress responses in *S. cerevisiae* are increased expression of genes encoding protein folding chaperones, proteins involved in respiration and enzymes for utilization of alternative carbon sources [28]; activation of Hog1 and mitogen-activated protein kinase (MAPK)-related pathways, including cell-wall remodelling [29]; changes in transcription rates and in mRNA stability [30]; and triggering of calcineurin-activated gene expression [31]. Looking across different yeasts, the integration of transcriptomic, proteomic and metabolomic data can help provide a complete picture of the stress response landscape [20, 32, 33]. Such omics studies indicate that there does not appear to be a single evolutionarily conserved thermotolerance mechanism; for example, a study of *K. marxianus* and *O. polymorpha* failed to find any similar patterns [34]. Specifically in *K. marxianus*, other studies comparing responses at 30 °C versus 45 °C found that *K. marxianus* presents a multi-faceted response to high temperatures, including a reduction in central metabolic activity, increased protein turnover and DNA repair [35], upregulation of the mitochondrial respiratory chain genes, and downregulation of glycolytic genes [36].

In a previous study, we carried out a large-scale comparative transcriptomic and proteomic analysis of *S. cerevisiae*, *K. marxianus* and *Y. lipolytica* growing under low pH, high temperature and high osmotic pressure [37]. All experiments were carried out in chemostats at a constant growth rate, thereby assessing long-term adaption to these stressful conditions rather than the short-term response to fluctuating stresses. The two major findings were, first, there is little commonality in how each yeast responds to the same stress; and second, evolutionarily young genes were over-represented in the sets of genes changing expression under adverse conditions. These indicate that genus- or species-specific genes that change expression are likely to be important in the physiological changes necessary for growth in harsh environments and thus these genes could be very useful for modifying yeasts for biotechnological processes where the growth medium and conditions are often suboptimal. In this current study, we focused on the evolutionary young genes that responded to elevated temperature in *K. marxianus* to determine their importance for the higher growth temperature of this yeast. To do this, using an updated *K. marxianus* genome annotation [38], we re-ran both the bioinformatic pipeline to identify this cohort of genes and the transcriptomic analysis to identify differentially expressed genes. We identified 12 *K. marxianus-*specific genes with increased expression at high temperature and successfully inactivated 11 of these. These mutants were assessed for growth at higher temperature, revealing that one of these genes, *KLMX_70834*, was specifically required for competitive growth at 45 °C. The protein encoded by *KLMX_70834* is unique to *K. marxianus* but possesses structural features that may be suggestive of RNA binding activity. As well as identification of this novel protein that is required for high-temperature growth in *K. marxianus*, our study validates the strategy of focusing on evolutionarily young genes to investigate niche adaptation in yeast.

## Methods

### Strains and cultivation

All *K. marxianus* strains used in this study are listed in Table 1. Knock-out mutants ΔT1 to ΔT13 were constructed in wild-type *K. marxianus* NBRC 1777 and the Δ*KLMX_70384* strain was constructed in the NHEJ-negative derivative, *K. marxianus* NBRC 1777 *dnl4*. Yeast strains were routinely grown on YPD medium (2 % peptone, 1 % yeast extract, 2 % dextrose) at 30 °C. Hygromycin B (200 µg ml^−1^) and G418 (200 µg ml^−1^) purchased from Sigma Aldrich were used for selection where required. For the drop tests, strains were grown overnight in 10 ml sterile water, harvested, washed and resuspended at an *A*_600nm_ of 1. Five micolitres of 1 :10 serial dilutions (until 10^−5^) of each strain was spotted onto agar plates, which were incubated at the appropriate temperature for 24 h. For screening of growth of multiple mutant strains at high temperature in liquid medium, a BioLector I (M2P-Labs) microfermentation system was used. For this, cells were grown overnight at 30 °C in YPD medium; the following morning, the cells were diluted to an *A*_600nm_ of 0.1 in fresh medium and 800 µl of each strain was loaded in a BioLector 48-well flower plate. A programme was run with the following settings: temperature=46.5 °C, biomass filter=Ex_620nm_ EM_620nm_, gain=20, shaking=1400 rpm. *Escherichia coli* strain DH5α was used for cloning purposes. The strain was maintained in LB medium (per litre: 5 g yeast extract, 10 g bactopeptone, 10 g NaCl) supplemented with 100 µg ampicillin ml^−1^ when required.

**Table 1.** Strains used in this study

Strain	Genotype	DMKU3-1042 gene ID	Mutation coordinates NBRC1777 genome	Source
NBRC 1777	WT			NITE Biological Research Centre, Japan
NBRC 1777 *dnl4*	*Δdnl4*			[42]
*Δhgt*	Δkmar_10531, Δkmar_10530, Δkmar_10529, Δkmar_10528, Δkmar_10527		HGT: CHR I Δ1,131,464–1,145,439	[42]
ΔT1	Δkmar_10001	KLMX_10556	CHR I ΔA 1,146,757	This study
ΔT2		KLMX_10792	CHR I Δ1,624,363–1,624,367	This study
ΔT4		KLMX_50030	CHR V ΔG 59,582	This study
ΔT5		KLMX_60003	CHR VI ΔC 8,314	This study
ΔT6	Δkmar_60126	KLMX_60133	CHR VI ΔC 282,973	This study
ΔT7	Δkmar_60261	KLMX_60270	CHR VI ΔG 563,131	This study
ΔT8		KLMX_60369	CHR VI ΔA 778,443	This study
ΔT9		KLMX_70384	CHR VII ΔA 807,181	This study
				
ΔT10		KLMX_70441	CHR VII ΔA 931,070	This study
ΔT11		KLMX_80304	CHR VIII Δ676,409–676,411	This study
ΔT13		KLMX_10646	CHR I Δ1,311,008–1,311,010	This study
*ΔKLMX_70384*	*dnl4 ΔKLMX_70384*		CHR VII Δ806,975–807,369	This study
ΔT9 I5: *KLMX_70384*	ΔT9 ΔI5: KLMX_70384compl		Insertion in I5 (chromosome IV: 240017–241741)	This study

### Differential expression and orthology analyses

Differential gene expression (DE) analysis was performed on the previously published RNA-seq dataset of strain CBS 6556 [37]. The RNA-seq data are available at SRA accession PRJNA531619. RNA-seq reads were aligned to the *K. marxianus* DMKU3-1042 genome [35], then assigned to genes using an updated genome annotation of the DMKU3-1042 genome available on the GWIPs-viz genome browser (Riboseq.org). DE analysis between the standard (Std) and high-temperature (HiT) condition, and orthologous inference to identify *K. marxianus*-specific genes, was performed using HISAT2 and Stringtie followed by the R-scripts contained within the OrthOmics package [37]. FASTA proteome files for orthology inference were obtained from uniprot.org. To check for potential roles of the target genes in different stress responses, a separate DE analysis was carried out on all *K. marxianus* RNA-seq datasets including low pH (lowpH) and osmotic stress (Osm) conditions. Genome alignments and reads per gene calling was performed using Bowtie2 [39], Samtools [40] and featureCounts [41], followed by the edgeR scripts available at https://rnnh.github.io/bioinfo-notebook/docs/DE_analysis_edgeR_script.html. For DE analysis, genes exhibiting a false discovery rate (FDR)<0.01 and log_2_ fold change (FC) >1 were considered significantly regulated. Normalized counts per million were calculated adjusting for library size according to the Trimmed Mean of the M-values (TMM) normalization method. A table of equivalence for gene IDs used in this study to perform the DE analysis, and the gene IDs of the published annotation [38], is available in Table S1 (available in the online version of this article).

### Molecular techniques

Mutants were constructed in WT strain NBRC 1777 by non-homologous end joining (NHEJ)-mediated gene inactivation, using the CRISPR-Cas9 system previously described [13, 42]. A detailed step by step protocol outlining these methods was recently published [43]. In brief, targeting sequences [for guide RNAs (gRNAs)] were ordered as DNA primers and inserted into plasmid pUCC001 by Golden Gate assembly. All plasmids and primers used in this study are listed in Tables 2 and 3, respectively. In the case of genes where an NBRC 1777 Gene ID was available, gRNA primers were designed using the sgRNA software [43, 44]. The remaining gRNAs were designed using the CRISPRdirect software [45], with the *K. marxianus* NBRC 1777 genome as template. Plasmids were introduced into *K. marxianus* using the standard LiAc/SS carrier DNA/PEG method with selection for transformants on Hygromycin B (200 µg ml^−1^). Transformants were screened for indel or frameshift mutations in the target gene using PCR amplification with diagnostic primers, followed by DNA sequencing. This method was successful in generating inactivating mutations in 11/12 target genes. The precise coordinates of deletion are listed in Table 1, using the genome coordinates from strain NBRC 1777 [46]. To allow for CRISPR-Cas9 plasmid loss, the mutant strains were grown overnight in 10 ml of YPD without antibiotic selection. Single colonies were tested on YPD with Hygromycin B to confirm lack of growth and hence loss of plasmid. Complementation of ΔT9 was achieved via chromosomal insertion of an expression cassette, containing the *KLMX_70384* coding sequence (CDS) under the control of the constitutive promoter p*PDC1* and the t*INU1* terminator. The expression cassette contains a KanMX selection marker and 850 bp homology arms for integration into the I5 intergenic region in chromosome IV [42]. The pI5-KLMX_70384compl plasmid (Table 3) was constructed via Golden Gate assembly of the *KLMX_70384* CDS, with the regulatory parts PDC1pr/P2 and INU1t/T1 into the pI5-MTU-DO-G418 plasmid using previously described methods [42]. For transforming into *K. marxianus* ΔT9, 1 µg of pI5-KLMX_70384compl was digested with *Sgs*I at 37 °C for 1 h. The digested product was transformed into the strain, with selection for transformants on G418. Colonies were screened via colony PCR with I5 locus-specific primers to identify positive transformants, and the integrity of the insert was confirmed by sequencing. A full ORF disruption of *KLMX_70384* was achieved in the *dnl4* derivative of *K. marxianus* NBRC 1777 using the same CRISPR-Cas9 system, this time taking advantage of the intrinsic homology dependent repair (HDR) apparatus. A 160 bp repair fragment was built by designing two primers Tar12_RF_F and Tar12_RF_R that shared 18 bp overlap and had 81 and 59 bp of homology to the 5′ and 3′ regions flanking *KLMX_70804*, respectively. The 5′ and 3′ homology arms were annealed and amplified via overlap extension PCR using Q5 High-Fidelity DNA Polymerase (New England Biolabs). Successful construction of the repair fragment was confirmed by gel electrophoresis and 1 µg of repair fragment was co-transformed with the CRISPR-Cas9 plasmid carrying the *KLMX_70384*-specific gRNA, into strain NBRC 1777 *dnl4*. Mutants were screened for via colony PCR, using primers Tar12_dia_F and Tar12_dia_R. *KLMX_70804* gene deletion was confirmed from the amplicon size of the PCR product (WT allele=798 bp; deleted allele=403 bp).

**Table 2.** Plasmids used in this study

Plasmid name	Relevant characteristics	Source
pUCC001	Modified from pUDP002 [13] for easy cloning of new guide RNA (gRNA) targets by Golden Gate assembly	[42]
pI5-MTU-DO-G418	Integrative plasmid modified from [42] with GFP drop-out, kanMX, targeting integration site I5 (chromosome IV: 240017–241741)	[19]
pI5-KLMX_70384compl	pPDC1-KLMX_70384-INU1t cloned into pI5-MTU-DO-G418	This study

**Table 3.** Primers used in this study

Primer name		Sequence	Relevant genes
**gRNA primers**			
Tar_1_gg_F		cgtcgcactcgaaccttataatag	KLMX_10556
Tar_1_gg_R		aaacctattataaggttcgagtgc	
Tar_2_gg_F		cgtcaacgtttttaaataattaaa	KLMX_10792
Tar_2_gg_R		aaactttaattatttaaaaacgtt	
Tar_4_gg_F		cgtcgtccttaattgtcactatgg	KLMX_50030
Tar_4_gg_R		aaaccaagagcagctgccgccaga	
Tar_5_gg_F		cgtctttcttctatctcacttcac	KLMX_60003
Tar_5_gg_R		aaacgtgaagtgagatagaagaaa	
Tar_6_gg_F		cgtctgtggtatacagcgatccac	KLMX_60133
Tar_6_gg_R		aaacgtggatcgctgtataccaca	
Tar_7_gg_F		cgtcacatgtagttcttgtgctcc	KLMX_60270
Tar_7_gg_R		aaacggagcacaagaactacatgt	
Tar_8_gg_F		cgtcgataaggtttcagcggatag	KLMX_60369
Tar_8_gg_R		aaacctatccgctgaaaccttatc	
Tar_9_gg_F		cgtctgctgaaacctctggtaaga	KLMX_70384
Tar_9_gg_R		aaactcttaccagaggtttcagca	
Tar_10_gg_F		cgtcgtgattagcgcttataactc	KLMX_70441
Tar_10_gg_R		aaacgagttataagcgctaatcac	
Tar_11_gg_F		cgtcgttctcttggggtaccccag	KLMX_80304
Tar_11_gg_R		aaacctggggtaccccaagagaac	
Tar_12_gg_F		cgtctcagggatgacaattattct	KLMX_10646
Tar_12_gg_R		aaacagaataattgtcatccctga	
**Diagnostic primers to confirm mutations**			
Tar_1_dia_F		tagaggaggtagatgtagcgg	KLMX_10556
Tar_1_dia_R		atgattccgtgaagccg	
Tar_2_dia_F		ggaaatgcgttagaaatgcttc	KLMX_10792
Tar_2_dia_R		caatgtactaacagggagca	
Tar_4_dia_F		ataaacggcagaatccgtt	KLMX_50030
Tar_4_dia_R		ggctgtgattaaaaagcact	
Tar_5_dia_F		catgtcattctcttacttaaccag	KLMX_60003
Tar_5_dia_R		aactttctccagatcaaatgaac	
Tar_6_dia_F		gcgtgtgttatattgtgttcg	KLMX_60133
Tar_6_dia_R		tcaccagaaagcagcatct	
Tar_7_dia_F		gtgtgcttacaatagcatagcac	KLMX_60270
Tar_7_dia_R		tccagtaaaaacaactacagagaa	
Tar_8_dia_F		atctgccaaattctccatg	KLMX_60369
Tar_8_dia_R		ctgagggttgatccttcac	
Tar_9_dia_F		cttctctaaactgctctgtct	KLMX_70384
Tar_9_dia_R		aagagcacagcggctaat	
Tar_10_dia_F		gaggaaatgaagaggtctttg	KLMX_70441
Tar_10_dia_R		ttcgtactttgtattctaggtttcc	
Tar_11_dia_F		ggtttggtttcccattc	KLMX_80304
Tar_11_dia_R		ctctacttcccaccattcc	
Tar_12_dia_F		attatgatatgaaagagaagcgc	KLMX_10646
Tar_12_dia_R		atctgtacgggaatgaaaa	
**Complementation**			
Tar9_GG_F	gcatcgtctcatcggtctcatatgatgtctgacaaggtcgaaga		
Tar9_GG_R	atgccgtctcaggtctcaggatttagttgatcaacttcttgaacttagca		
I5US_F	agtagtgagtgacagacac		
P2R	gcaattatttggtttgggtgtg		
**Repair fragment**			
Tar9_RF_F	gaaaactagttccatatagtatcccattattactcatttctctcttgttagctcgtattccagccaagcaaacgaaaagtccgtcgttacttaca		
Tar9_RF_R	agatagattagattaattaattaattattaagtattatgggaattagaagactaaggatgtagtgtaagtaacgacg		
			

### Competition experiment

The *ΔKLMX_70384* and parental strains were grown individually overnight in 10 ml of YPD broth at 30 °C. The following morning, they were mixed at a 50 : 50 ratio to a final *A*_600nm_ of 1, in a 50 ml falcon tube. The co-culture was then diluted to a final *A*_600nm_ of 0.1 into six 250 ml flasks, in YPD, to a final volume of 50 ml. Three of the flasks were placed in a shaking incubator at 30 °C, and the remaining three were placed in a shaking water bath, pre-warmed at 45 °C. One millilitre of the co-culture was serially diluted and plated on YPD plates, for colony screening at time=0 (T0). The six flasks were sampled at a regular interval of 12 h for 48 h, and 1 ml of the co-culture was serially diluted and plated on YPD plates. The plates were incubated for 24 h at 30 °C, and 10 random colonies were picked from them for screening via colony PCR, using primers Tar12_dia_F and Tar12_dia_R (Table 3). Strains were distinguished by the size of the amplicon (WT allele=798 bp; deleted allele=403 bp). The significance of the difference between the parental and *ΔKLMX_70384* strains in co-culture composition, between T0 and 24 h, was calculated with a paired *t*-test, with *P*<0.05 considered to be significant.

## Results

### Identification of 12 novel genes with a possible role in thermotolerance in *K. marxianus*

Using an updated annotation of the *K. marxianus* DMKU3-1042 genome [38], we mapped the RNA-seq reads of the standard growth condition (Std) and high temperature (HiT) datasets to identify DE genes. A total of 216 genes with significant changes in expression were identified, and these are listed in Table S2. As the updated annotation uses a different gene identifier (KLMX versus KMXK), the table also includes this information to facilitate comparison between the studies. We wished to know which of these were evolutionarily young and *K. marxianus*-specific. To do this, single copy protein-coding genes were identified and subjected to pairwise orthology inference using Orthofinder [47] against the proteomes of *K. lactis, S. cerevisiae, Y. lipolytica* and *Homo sapiens*. The yeast species were chosen because they span a wide range of the budding yeast subphylum (*Saccharomycetaceae*) [48] and *H. sapiens* represents a distantly related eukaryote.

**Fig. 1.**
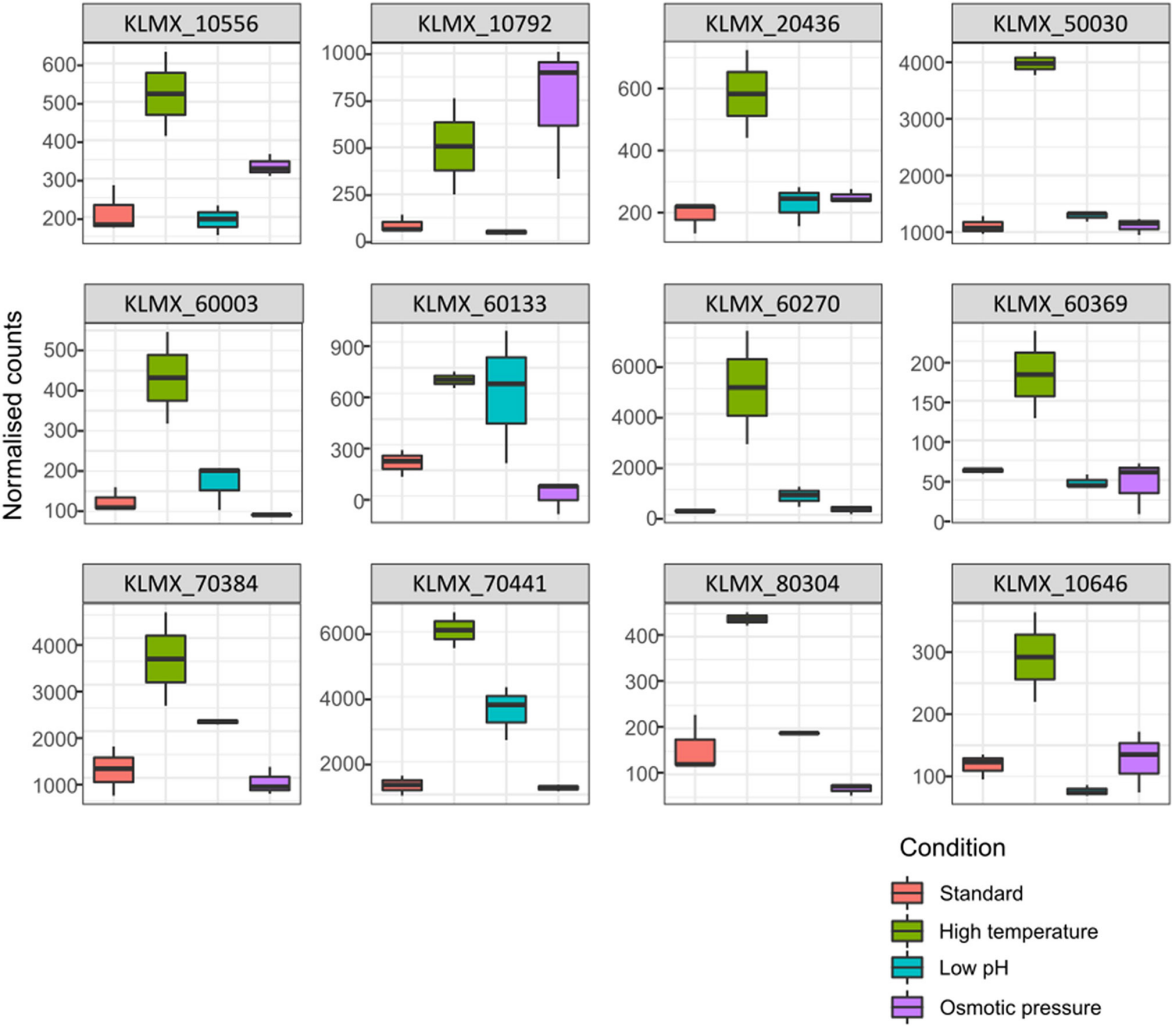
*K. marxianus*-specific genes are upregulated at higher temperature. Data show the expression of 12 *K. marxianus*-specific genes from chemostat cultures under different conditions. Boxplots showing normalized counts of target gene reads, representing relative abundance of transcripts at standard (30 °C, pink) versus high temperature (40 °C, green), low pH (3.5, blue) and high osmotic pressure (1 M KCl, purple). Normalized counts for the genes were calculated using TMM normalization in edgeR. Boxplot was obtained using ggplot2.

Following the hierarchical approach described by Doughty and colleagues [37], genes were segregated into five groups based on gene age, which was inferred by the presence/absence of orthologous proteins in the proteomes of the aforementioned species. Proteins only present in the final group, with no orthologues in any of the other proteomes, were considered to be encoded by evolutionarily young, *K. marxianus*-specific genes. Of the 55 genes in this group, 16 were found to be differentially expressed during high-temperature growth, with 13 upregulated and three downregulated (Table S2). To confirm that these genes really were *K. marxianus*-specific, a blastp search was individually performed with each protein sequence against the non-reduntant database which includes a wider range of species (Table S3). This led to exclusion of three candidate genes (one upregulated and two downregulated) that potentially had orthologues in other yeast species. Of the remaining 13 *K. marxianus*-specific genes, one, *KLMX_70441,* showed a significant hit (E-value: 9e-109, percantage identity: 71 %, query coverage: 97 %) with an NADPH-dependent oxidoreductase protein from the *Acinetobacter* species, suggesting acquisition by horizontal gene transfer (Fig. S1). This gene was auto-annotated as ‘*YwnB*’ [35] on the GWIPs-viz browser ([38, 49], reflecting homology to a *Bacillus subtilis* gene of unknown function. Two others, *KLMX_60133* (chitin synthesis regulation, E-value: 4e-122) and *KLMX_60270* (lysine-rich arabinogalactan protein, E-value: 7e-168), were suggested to have particular functional domains but no clear orthologues were identified. The percentage of DE genes in the *K. marxianus*-specific group (24 %) was higher than other orthogroups (2.4–13 %), consistent with previous findings of enrichment in differential expression of evolutionary young genes under adverse conditions [37]. As we were most interested in genes that might have potential for biotechnology, we focused on the 12 upregulated *K. marxianus*-specific genes (Table 4). Six of these are from the set of newly annotated genes and so were absent from any previous analyses and, although all 12 genes are present in genomes of strains CBS6556 and NBRC1777, only two (NBRC1777) or three (CBS6556) are annotated with gene identifiers in those genomes, and so we use the DMKU3-1024 gene IDs for subsequent analyses and discussion for all strains. Since the CDS of most of the 12 genes are short (63–1713 bp), there was the possibility that these genes could encode non-coding RNAs rather than proteins [50]. To assess this, we examined each gene individually on the GWIPs-viz browser [38], which displays data from ribosome profiling and a range of other transcriptomic techniques (Fig. S2). Despite two of the genes showing low ribo-seq reads, we confirmed that all 12 genes are translated and are therefore confirmed protein-coding genes. To determine whether the identified genes are only responsive to high-temperature stress, the DE analysis was repeated for the low pH and osmotic conditions used in the original study (Tables S4 and S5). The majority of genes were exclusively upregulated under high-temperature growth, though expression of one gene was also elevated under high osmotic pressure (Fig. 1).

**Table 4.** List of *K. marxianus*-unique genes upregulated at high temperature List of genes unique to *K. marxianus* species, upregulated at high temperature. Where available, corresponding NBRC 1777 and CBS 6556 strain gene IDs are provided. Functional annotation deriving from blast comparison is provided where available. Unknown=search on blastx database shows no similarity to any other entry, but just a match with *K. marxianus* chromosomes or gene of unknown function.

Gene ID DMKU3-1042	logFC	Gene ID NBRC1777	Gene ID CBS6556	Function
KLMX_10556*	1.44	na	na	Unknown
KLMX_10792*	2.59	na	na	Unknown
KLMX_20436*	1.56	na	na	Unknown
KLMX_50030*	1.82	na	na	Unknown
KLMX_60003*	2.30	na	na	Unknown
KLMX_60133†	1.19	KMAR_60126	KMXK_0F04470	Chitin synthesis regulation
KLMX_60270†	4.96	KMAR_60261	KMXK_0F03060	Lysine-rich arabinogalactan protein
KLMX_60369	1.55	na	na	Unknown
KLMX_70384*	1.53	na	na	Unknown
KLMX_70441	1.55	na	na	NADPH-dependent oxidoreductase
KLMX_80304	1.54	na	na	Unknown
KLMX_10646†	1.81	na	KMXK_0A02020	Unknown

*Previously unknown genes, identified by mapping the ribo-seq reads to the DMKU3-1042 strain genome [38].

†Genes previously reported as upregulated under high temperature in [37].

### Screening for the role of the unique upregulated genes in thermotolerance

**Fig. 2.**
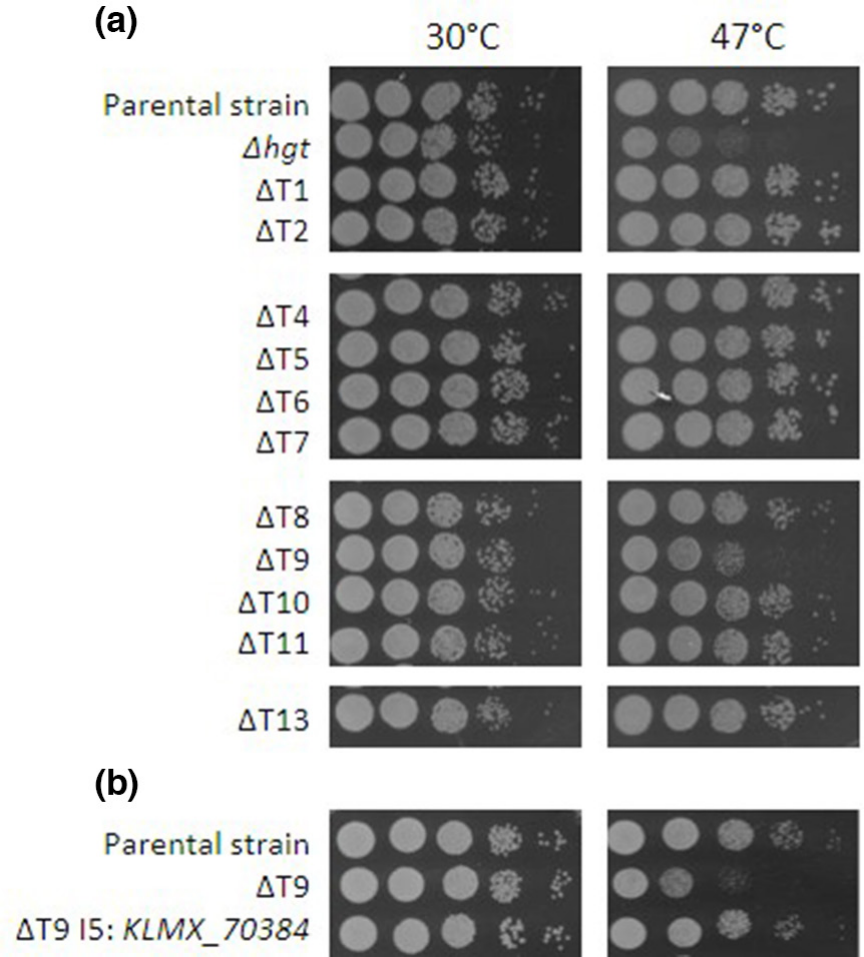
Assessment of growth of *K. marxianus* mutants at high temperature. (**a**) The target genes identified in this study were inactivated by nucleotide deletion in the parental strain. The resultant knock-out mutants were plated in serial dilution on YPD plates and grown at 30 and 47 °C for 24 h. (**b**) Complementation of the ΔT9 mutant via insertion of a KLMX_70384 expression cassette in the parental strain restores the phenotype.

To test whether these genes played a role in thermotolerance, 11 of the genes were individually inactivated using CRISPR-Cas9 and the endogenous NHEJ repair mechanism. Despite multiple efforts, we were unsuccessful in inactivating *KLMX_20436,* possibly because this gene may be essential. In each case, a deletion of one to four bases occurred, resulting in frameshift mutations that inactivate the genes (Table 1). These strains, labelled ΔT1–ΔT13, were assessed for growth on agar plates at 30 and 47 °C (Fig. 2). For comparison, the thermosensitive *Δhgt* strain was used [51]. Of the 11 mutants tested, only ΔT9, carrying a mutation in *KLMX_70384,* displays a growth-impaired phenotype after 24 h of growth at 47 °C. For complementation tests, an expression cassette containing the *KLMX_70384* CDS under a strong *PDC1* promoter was also constructed and integrated into the genome of ΔT9 (Fig. S3). Growth assays at 47 °C showed that the complemented strain recovered the parental phenotype, confirming that the temperature-sensitive phenotype in ΔT9 was due to inactivation of *KLMX_70384* (Fig. 2). As ΔT9 has a single point mutation that could revert, for later studies, a *ΔKLMX_70384* mutant with a precise deletion of the entire CDS was constructed in a *dnl4* background (lacking NHEJ) using CRISPR-Cas9 and the HDR machinery of *K. marxianus*.

**Fig. 3.**
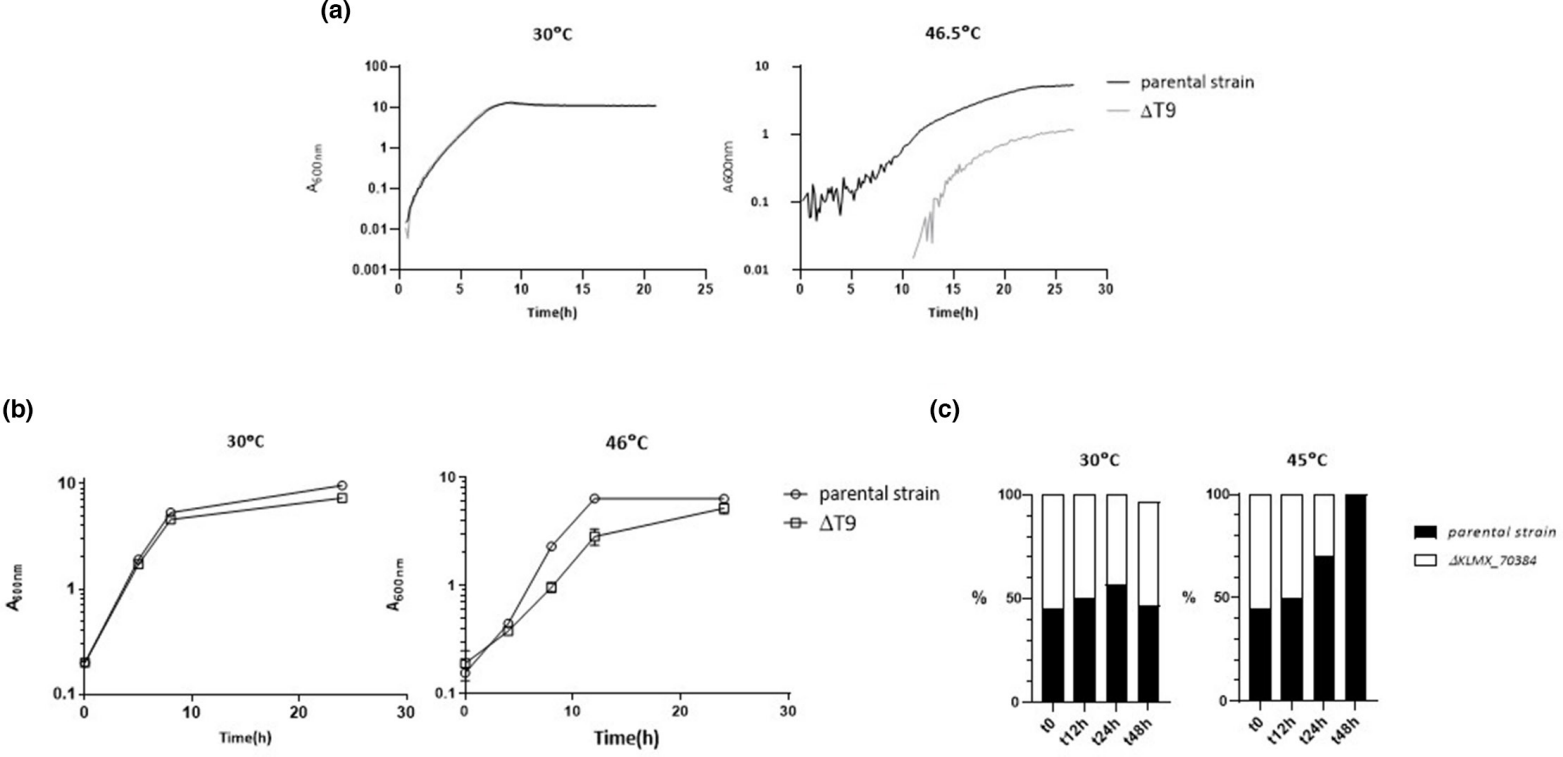
Deletion of KLMX_70804 impairs high-temperature adaptation of *K. marxianus*. (**a**) Growth curves comparing growth of *K. marxianus* parental and ΔT9 strains in YPD medium at low (30 °C) and high temperature (46.5 °C) using a Biolector microtitre plate system. *A*_600nm_ is reported in a log_10_ scale. The plot represents the average reads of four (30 °C) and three replicates (46.5 °C). (**b**) Growth comparison of parental and ΔT9 strains at 30 and 46 °C, individually grown in shake flasks. The *A*_600nm_ values represent the mean of four biological replicates and are plotted on a log_10_ scale. (**c**) A competition experiment was performed, in which a 50 : 50 co-culture of the parental strain and ΔKLMX_70384 strain was grown for 48 h at low (30 °C) and high (45 °C) temperature. The co-culture was sampled at the indicated times and serial dilutions were plated and incubated for 24 h. The proportion of parental and ΔKLMX_70384 strains was determined using diagnostic PCR amplification of 30 randomly selected colonies at each time point.

Several experiments were also performed to assess growth in liquid culture (Fig. 3). All 11 strains carrying the single point mutation were grown in a microtitre plate system again with only ΔT9 showing a temperature-sensitive phenotype, evident by an extended lag phase and lower *A*_600nm_ after 26 h than the parental strain (Fig. 3a, data just shown for ΔT9 and parental strain). A slower growth rate was also evident in flask culture, though the phenotype did not appear to be as pronounced as had been seen in the plate assay (Fig. 3b). Next, to investigate the possible benefit conferred by *KLMX_70384* at higher temperature, a competition experiment was performed (Fig. 3c). A co-culture containing equal cell number of the *ΔKLMX_70384* and parental strains was split and incubated at either 30 or 45 °C. The relative percentages of parental and mutant were determined by differential PCR after 12, 24 and 48 h of growth. Whereas the ratio of strains remained constant at 30 °C, the proportion of *ΔKLMX_70384* progressively decreased at 45 °C, showing a statistically significant effect after 24 h (*P*=0.012), and complete exclusion of *ΔKLMX_70384* after 48 h.

### Bioinformatic analysis of *KLMX_70384*

**Fig. 4.**
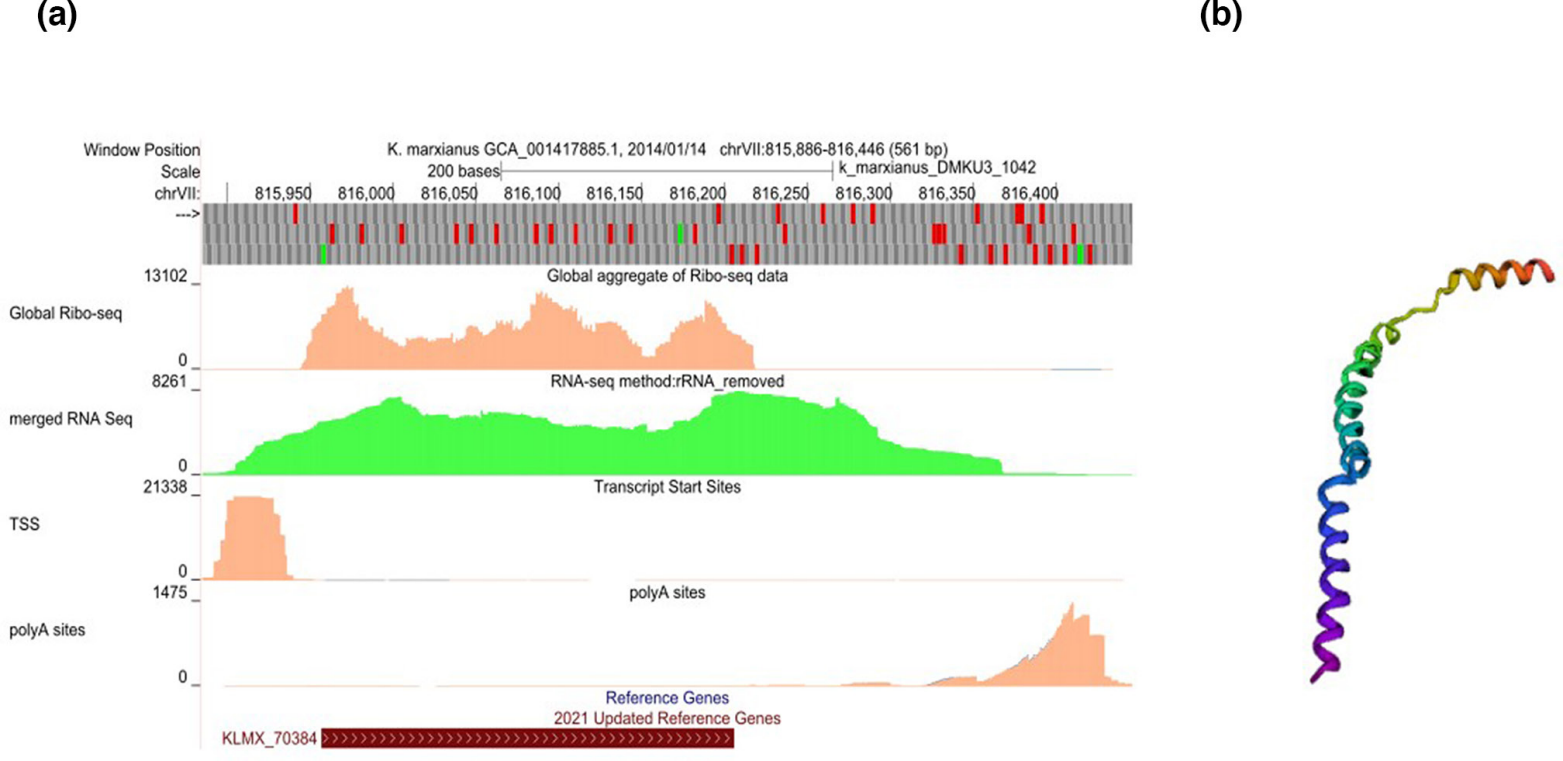
Transcription, translation and structure of KLMX_70384. (a) GWIPS-viz view of *KLMX_70384* showing, from top to bottom: global ribo-seq reads (scale: 0–13 102); merged RNA-seq reads (scale: 0–8261); transcription start sites (TSS, scale: 0–21338); polyA sites (scale: 0–1475). The CDS position is indicated by the red bar on the bottom track. (**b**) *Ab initio* prediction of the tertiary structure of the KLMX_70384 protein with Robetta. Structure prediction is predominantly α-helical. Colour in the figure is from blue (N terminus) to red (C terminus).

**Fig. 5.**
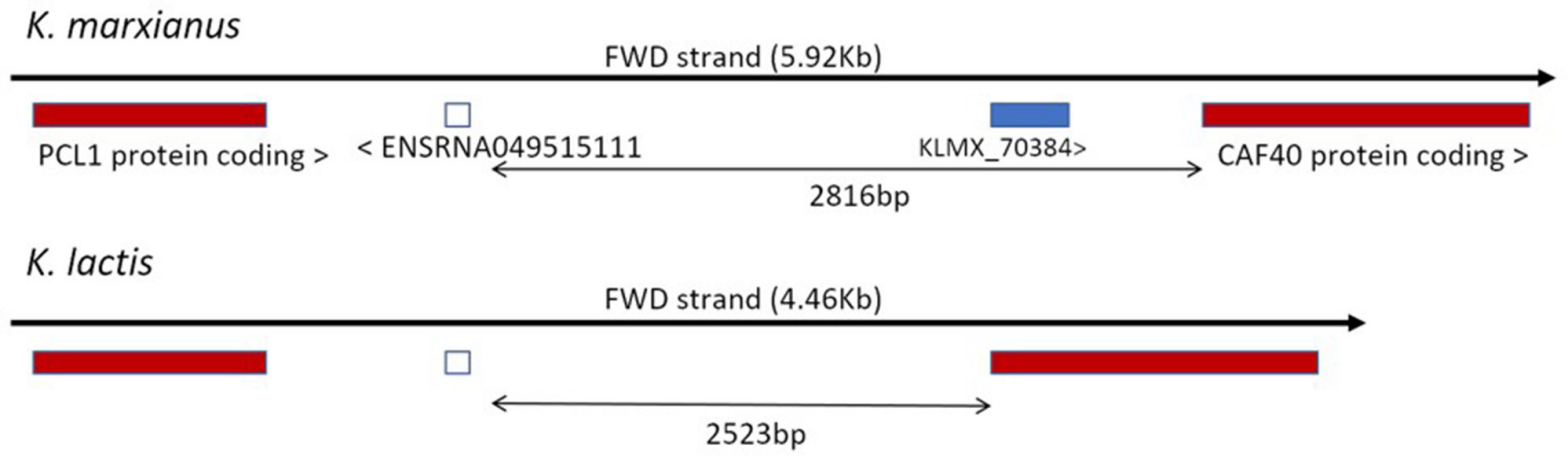
Genomic features of KLMX_70384. Comparison of KLMX_70384 genome locus between *K. marxianus* and *K. lactis*. Homologous flanking genes are reported in red, homologous Small nucleolar RNAs (snoRNAs) are represented by the white square, KLMX_70384’s CDS is represented by the blue square, and the non-homologous region between the SNR40 and CAF40 genes is indicated by the black arrow.

Comparison of available genome sequences of *K. marxianus* revealed that there are two allelic forms of *KLMX_70384*: a longer version (249 bp) predicted to encode an 83 aa peptide, and a shorter version (225 bp) lacking a 23 bp region which results in the loss of a region of the peptide between the amino acids Ser30 and Lys39, resulting in a predicted 75 aa peptide. There was no obvious pattern to which strains had each form, and to assess whether there was a functional difference, each of the versions was integrated into the genome of ΔT9. Both alleles of *KLMX_70384* complemented the thermosensitive phenotype, indicating that the lacking peptide region in the shorter version is not essential for the protein’s function (data not shown). In its longer version, *KLMX_70384* is predicted to encode an 83 aa peptide and its translation is confirmed by ribo-seq data (Fig. 4a). A blastp analysis did not find any orthologues in the database, and the only hit from a Pfam search [52] was a low, non-significant similarity (E-value: 0.06) to a domain of human *SART-1. SART-1* is the orthologue of *S. cerevisiae SNU66*, which is encoded by *KLMX_20322* in *K. marxianus*. *Ab initio* modelling using Robetta [53] predicts that KLMX_70384 forms a structure with three alpha-helices, oriented in a linear configuration (Fig. 4b). *KLMX_70384* is located within a 2816 bp region previously annotated as intergenic on chromosome VII, between the snoRNA40 gene (*ENSRNA049515111*) and *CAF40* (*KLMX_70385*) (Fig. 5). This entire region is conserved (90–100 %) in sequenced strains of *K. marxianus* but bears no homology to the equivalent 2523 bp intergenic region in the closest relative, *K. lactis*, nor to any other sequences in databases. Comparison on the Saccharomyces Genome Database finds that synteny is conserved in *S. cerevisiae* but in this case the intergenic region (which lacks a *KLMX_70384* homologue) contains the autonomously replicating sequence ARS1407 (yeastgenome.org).

## Discussion

The main aim of this study was to generate data to address the hypothesis put forward in a previous study that evolutionary young genes could be involved in adaption to growth under adverse conditions [37]. We did this by investigating genes that were upregulated during steady-state growth at high temperature in *K. marxianus*. The availability of an improved annotation of *K. marxianus* that added ~170 genes and corrected mis-annotation of many others using ribo-seq data [38] was an advantage, but it also required that we perform a complete *de novo* bioinformatic analysis of the RNA-seq data generated in our previous chemostat study [37]. The outcome of this analysis was the identification of 12 genes that were unique in *K. marxianus* and upregulated at 40 °C. We successfully mutagenized 11 of the 12 candidate genes to assess their role in high-temperature growth and found that inactivation of *KLMX_70384* displayed the predicted phenotype – normal growth at 30 °C but compromised growth at 45–47 °C. Interestingly, the mutant was completely outcompeted by its parent in a co-culture experiment, indicating that the gene is required for competitive growth in high-temperature niches. The other ten mutants did not exhibit decreased growth at higher temperatures, and future studies might identify changes using competition assays or double or triple mutants to reveal new interactions between genes. Our finding that *KLMX_70384* is required for competitive growth at elevated temperature is consistent with the hypothesis that was being tested.

Evolutionarily young genes generally are short, non-essential genes and this was also true of the genes that we identified: ten of the ORFs were between 28 and 225 aa, and *KLMX_20832* was the only gene that we failed to inactivate, possibly indicating that it is essential. We were not able to make any predictions regarding the function of KLMX_20832. *De novo* structure prediction with Robetta indicates that the protein forms an α-helix domain (0.86 confidence) but searches on the PFAM and blastp databases did not return any matches.

With such short genes, there is always a concern that they are either not expressed or encode non-coding RNAs, but we confirmed using ribosome profiling data that all 12 are translated, and thus are genuine protein-encoding genes. This shows the utility of the *K. marxianus* ribosome profiling data in the GWIPs-viz genome browser (Riboseq.org), a resource that is only available for a few yeast species [49]. Of the 12 genes, one shows evidence of having been acquired by horizontal gene transfer, and it was only possible to predict two putative functional protein domains in the others, illustrating the challenge of working with novel genes. The overall lack of functional annotation for the identified genes reflects the limitation of homology-based algorithms for genome annotation [54], especially when it comes to species phylogenetically distant from the extensively annotated *S. cerevisiae* [55]. This matches the experience of previous studies with *K. marxianus*, *Y. lipolytica* and *Lachancea kluyveri* [37, 56]. *KLMX_70384* codes for a relatively short protein (83 aa) when compared with the average *K. marxianus* protein length of 500 aa [35]. In yeast, proteins below 90 aa are classified as small proteins [57] and are enriched for seven biochemical functions: structural constituent of ribosomes, pre-mRNA splicing factor, ubiquitin-conjugating enzyme, cytochrome-C oxidase, thiol-disulphide exchange and tubulin binding. The low similarity to the splicing-associated protein *SART-1*/*SNU66*, as well as its predicted linear three-helix structure, suggests that KLMX_70384 may be an RNA-binding protein, possibly associated with the RNA processing or splicing machinery in some way (58 [59]. In this regard, it is notable that a group of deletion mutants for mRNA processing genes display a thermosensitive phenotype in *S. cerevisiae* [60].

The origins of species-specific genes remain unclear with different possible mechanisms postulated. With the exception of *KLMX_ 70441,* the absence of any identified homologues in our gene set suggests that the main source is *de novo* evolution rather than acquisition by horizontal gene transfer, gene duplication or recombination. The clue to the birth of *KLMX_70384* may lie with its location within a region of the genome previously annotated as intergenic. Notably, in *S. cerevisiae*, this intergenic region hosts an Autonomously replicating sequences (ARS) which, however, shares no similarity with the sequence of *KLMX_70384*. Intergenic regions in yeast are more prone to evolution and mutation, and hence new coding sequences are more likely to originate from them [61]. A similar mechanism of evolution, although rare [62], has been described before in yeast [63]. We previously suggested a scenario for evolution of genes that confer an advantage under harsh conditions [37]. In that model, mutations regularly arise but those that occur in ancient genes, typically associated with core processes, are more likely to be detrimental and lost. Young genes, which are typically not required under standard growth conditions, can better tolerate mutations and so pools of mutants build up. When conditions become adverse, those rare mutations that confer a growth advantage are selected. In this way, young genes become important for adaptation to new niches. Based on our data, we speculate that the emergence of *KLMX_70384* played some role in helping *K. marxianus* adapt to growth at higher temperatures, a trait that is absent in all other *Kluyveromyces* species. It also helps explain why a conserved thermotolerance mechanism is not found in other yeasts, such as *O. polymorpha*, since it implies that different species-specific genes will be involved.

While understanding evolutionary processes is fascinating, part of the rationale for studying thermotolerance is to identify genes and processes that can be used to improve yeasts for biotechnology. One hope is that it may be possible to engineer thermotolerance into mesophilic yeasts by heterologous expression of single genes. We tested whether expression of *KLMX_70384* in *K. lactis* would improve the thermotolerance of this yeast, but it did not (data not shown). This is probably not surprising since it is generally considered that there are multiple requirements for higher temperature growth and thus overcoming one hurdle will not be enough. It is also possible that a protein such as KLMX_70384 will have specific interactions that only take place with other proteins that share an evolutionary history. Despite this, it is still valuable to try to understand what processes are required to function at higher temperature as this could identify alternative routes to strain improvement. While not definitive, the indications are that KLMX_70384 could be involved in RNA processing and further suggest that this is an area worth further investigation.

## Data availability

The RNA-seq dataset analysed in this study can be retrieved under SRA accession PRJNA531619 [https://www.ncbi.nlm.nih.gov/bioproject/PRJNA531619/]. DE analysis and OrthoFinder scripts used to analyse the standard and the high-temperature RNA-seq datasets are available at the OrthOmics page at https://github.com/SysBioChalmers/OrthOmics. The script used to compare all the stress datasets can be found at https://rnnh.github.io/bioinfo-notebook/docs/DE_analysis_edgeR_script.html. DE analysis tables generated in this study can be found in Tables S2 and S4. *K. marxianus* genome accession numbers are PRJDA65233 (DMKU3-1042) and SRX3541357 (NBRC 1777). The updated DMKU3-1042 annotation is publicly available on the GWIPs-viz genome browser (Riboseq.org).

### Statement on author approval

As corresponding author on the paper, and Principal Investigator on the project, I approve publication of this paper on behalf of Iván Domenzain.

## Supplementary Data

Supplementary material 1

Supplementary material 2

Supplementary material 3

Supplementary material 4

Supplementary material 5

Supplementary material 6

## References

[1] Lange L, Connor KO, Arason S, Bundgård-Jørgensen U, Canalis A (2020). Developing a Sustainable and Circular Bio-Based Economy in EU: By Partnering Across Sectors, Upscaling and Using New Knowledge Faster, and For the Benefit of Climate, Environment & Biodiversity, and People & Business. Front Bioeng Biotechnol.

[2] Patra P, Das M, Kundu P, Ghosh A (2021). Recent advances in systems and synthetic biology approaches for developing novel cell-factories in non-conventional yeasts. Biotechnol Adv.

[3] Rebello S, Abraham A, Madhavan A, Sindhu R, Binod P (2018). Non-conventional yeast cell factories for sustainable bioprocesses. FEMS Microbiol Lett.

[4] Rodicio R, Heinisch JJ (2013). Yeast on the milky way: genetics, physiology and biotechnology of *Kluyveromyces lactis*. Yeast.

[5] Zeng S, Liu H, Shi T, Song P, Ren L (2018). Recent advances in metabolic engineering of *Yarrowia lipolytica* for lipid overproduction. Eur J Lipid Sci Technol.

[6] Castro RCA, Roberto IC (2014). Selection of a thermotolerant *Kluyveromyces marxianus* strain with potential application for cellulosic ethanol production by simultaneous saccharification and fermentation. Appl Biochem Biotechnol.

[7] Phithakrotchanakoon C, Phaonakrop N, Roytrakul S, Tanapongpipat S, Roongsawang N (2020). Protein secretion in wild-type and Othac1 mutant strains of thermotolerant methylotrophic yeast *Ogataea thermomethanolica* TBRC656. Mol Biol Rep.

[8] Cankorur-Cetinkaya A, Narraidoo N, Kasavi C, Slater NKH, Archer DB (2018). Process development for the continuous production of heterologous proteins by the industrial yeast, *Komagataella phaffii*. Biotechnol Bioeng.

[9] Manfrão-Netto JHC, Gomes AMV, Parachin NS (2019). Advances in using *Hansenula polymorpha* as chassis for recombinant protein production. Front Bioeng Biotechnol.

[10] Puseenam A, Kocharin K, Tanapongpipat S, Eurwilaichitr L, Ingsriswang S (2018). A novel sucrose-based expression system for heterologous proteins expression in thermotolerant methylotrophic yeast *Ogataea thermomethanolica*. FEMS Microbiol Lett.

[11] Zhang B, Zhao X, Wang Z, Wang H, Zhou J (2021). Efficient secretory expression and purification of food-grade porcine myoglobin in *Komagataella phaffii*. J Agric Food Chem.

[12] Cai P, Gao J, Zhou Y (2019). CRISPR-mediated genome editing in non-conventional yeasts for biotechnological applications. Microb Cell Fact.

[13] Juergens H, Varela JA, Gorter de Vries AR, Perli T, Gast VJM (2018). Genome editing in *Kluyveromyces* and Ogataea yeasts using a broad-host-range Cas9/gRNA co-expression plasmid. FEMS Yeast Res.

[14] Li J, Rong L, Zhao Y, Li S, Zhang C (2020). Next-generation metabolic engineering of non-conventional microbial cell factories for carboxylic acid platform chemicals. Biotechnol Adv.

[15] Yang Z, Blenner M (2020). Genome editing systems across yeast species. Curr Opin Biotechnol.

[16] Donzella L, Varela J A, Sousa M J, and Morrissey J P (2021). Identification of Novel Pentose Transporters in Kluyveromyces Marxianus Using a New Screening Platform. FEMS Yeast Res.

[17] Nurcholis M, Lertwattanasakul N, Rodrussamee N, Kosaka T, Murata M (2020). Integration of comprehensive data and biotechnological tools for industrial applications of *Kluyveromyces marxianus*. Appl Microbiol Biotechnol.

[18] Karim A, Gerliani N, Aïder M (2020). *Kluyveromyces marxianus*: An emerging yeast cell factory for applications in food and biotechnology. Int J Food Microbiol.

[19] Rajkumar AS, Morrissey JP (2020). Rational engineering of *Kluyveromyces marxianus* to create a chassis for the production of aromatic products. Microb Cell Fact.

[20] Li P, Fu X, Chen M, Zhang L, Li S (2019). Proteomic profiling and integrated analysis with transcriptomic data bring new insights in the stress responses of *Kluyveromyces marxianus* after an arrest during high-temperature ethanol fermentation. Biotechnol Biofuels.

[21] Yu Y, Mo W, Ren H, Yang X, Lu W (2021). Comparative genomic and transcriptomic analysis reveals specific features of gene regulation in *Kluyveromyces marxianus*. Front Microbiol.

[22] Gong Z, Nielsen J, Zhou YJ (2017). Engineering robustness of microbial cell factories. Biotechnol J.

[23] Jiang T, Li C, Teng Y, Zhang R, Yan Y (2020). Recent advances in improving metabolic robustness of microbial cell factories. Curr Opin Biotechnol.

[24] Madeira-Jr JV, Gombert AK (2018). Towards high-temperature fuel ethanol production using *Kluyveromyces marxianus*: On the search for plug-in strains for the Brazilian sugarcane-based biorefinery. Biomass and Bioenergy.

[25] Malairuang K, Krajang M, Rotsattarat R, Chamsart S (2020). Intensive multiple sequential batch simultaneous saccharification and cultivation of *Kluyveromyces marxianus* SS106 thermotolerant yeast strain for single-step ethanol fermentation from raw cassava starch. Processes.

[26] Suzuki T, Hoshino T, Matsushika A (2019). High-temperature ethanol production by a series of recombinant xylose-fermenting *Kluyveromyces marxianus* strains. Enzyme Microb Technol.

[27] Yanase S, Hasunuma T, Yamada R, Tanaka T, Ogino C (2010). Direct ethanol production from cellulosic materials at high temperature using the thermotolerant yeast *Kluyveromyces marxianus* displaying cellulolytic enzymes. Appl Microbiol Biotechnol.

[28] Gasch AP, Spellman PT, Kao CM, Carmel-Harel O, Eisen MB (2000). Genomic expression programs in the response of yeast cells to environmental changes. Mol Biol Cell.

[29] Leach MD, Budge S, Walker L, Munro C, Cowen LE (2012). Hsp90 orchestrates transcriptional regulation by Hsf1 and cell wall remodelling by MAPK signalling during thermal adaptation in a pathogenic yeast. PLoS Pathog.

[30] Castells-Roca L, García-Martínez J, Moreno J, Herrero E, Bellí G (2011). Heat shock response in yeast involves changes in both transcription rates and mRNA stabilities. PLoS One.

[31] Kraus PR, Heitman J (2003). Coping with stress: calmodulin and calcineurin in model and pathogenic fungi. Biochem Biophys Res Commun.

[32] Shui W, Xiong Y, Xiao W, Qi X, Zhang Y (2015). Understanding the mechanism of thermotolerance distinct from heat shock response through proteomic analysis of industrial strains of *Saccharomyces cerevisiae*. Mol Cell Proteomics.

[33] Walther D, Strassburg K, Durek P, Kopka J (2010). Metabolic pathway relationships revealed by an integrative analysis of the transcriptional and metabolic temperature stress-response dynamics in yeast. OMICS.

[34] Lehnen M, Ebert BE, Blank LM (2019). Elevated temperatures do not trigger a conserved metabolic network response among thermotolerant yeasts. BMC Microbiol.

[35] Lertwattanasakul N, Kosaka T, Hosoyama A, Suzuki Y, Rodrussamee N (2015). Genetic basis of the highly efficient yeast *Kluyveromyces marxianus*: complete genome sequence and transcriptome analyses. Biotechnol Biofuels.

[36] Fu X, Li P, Zhang L, Li S (2019). Understanding the stress responses of *Kluyveromyces marxianus* after an arrest during high-temperature ethanol fermentation based on integration of RNA-Seq and metabolite data. Appl Microbiol Biotechnol.

[37] Doughty TW, Domenzain I, Millan-Oropeza A, Montini N, de Groot PA (2020). Stress-induced expression is enriched for evolutionarily young genes in diverse budding yeasts. Nat Commun.

[38] Fenton DA, Kiniry SJ, Yordanova MM, Baranov PV, Morrissey JP Development of a ribosome profiling protocol to study translation in *Kluyveromyces marxianus*.

[39] Langmead B, Salzberg SL (2012). Fast Gapped-Read Alignment with Bowtie 2. http://bowtie-bio.sourceforge.net/bowtie2/index.shtml.

[40] Danecek P, Bonfield JK, Liddle JM, Ohan J, Pollard V (2021). Twelve years of SAMtools and BCFtools. GigaScience.

[41] Yang L, Smyth GK, Shi W (2014). Sequence Analysis FeatureCounts: An Efficient General Purpose Program for Assigning Sequence Reads to Genomic Features. http://subread.sourceforge.net.

[42] Rajkumar AS, Varela JA, Juergens H, Daran JMG, Morrissey JP (2019). Biological parts for *Kluyveromyces marxianus* synthetic biology. Front Bioeng Biotechnol.

[43] Rajkumar AS, Morrissey JP (2022). Protocols for marker-free gene knock-out and knock-down in *Kluyveromyces marxianus* using CRISPR/Cas9. FEMS Yeast Res.

[44] Xie S, Shen B, Zhang C, Huang X, Zhang Y (2014). sgRNAcas9: a software package for designing CRISPR sgRNA and evaluating potential off-target cleavage sites. PLoS One.

[45] Naito Y, Hino K, Bono H, Ui-Tei K (2015). CRISPRdirect: software for designing CRISPR/Cas guide RNA with reduced off-target sites. Bioinformatics.

[46] Inokuma K, Ishii J, Hara KY, Mochizuki M, Hasunuma T (2015). Complete genome sequence of *Kluyveromyces marxianus* NBRC1777, a nonconventional thermotolerant yeast. Genome Announc.

[47] Emms DM, Kelly S (2015). OrthoFinder: solving fundamental biases in whole genome comparisons dramatically improves orthogroup inference accuracy. Genome Biol.

[48] Shen X-X, Opulente DA, Kominek J, Zhou X, Steenwyk JL (2018). Tempo and mode of genome evolution in the budding yeast subphylum. Cell.

[49] Michel AM, Fox G, M Kiran A, De Bo C, O’Connor PBF (2014). GWIPS-viz: development of a ribo-seq genome browser. Nucleic Acids Res.

[50] Parker S, Fraczek MG, Wu J, Shamsah S, Manousaki A (2018). Large-scale profiling of noncoding RNA function in yeast. PLoS Genet.

[51] Varela JA, Puricelli M, Montini N, Morrissey JP (2019). Expansion and diversification of MFS transporters in *Kluyveromyces marxianus*. Front Microbiol.

[52] Mistry J, Chuguransky S, Williams L, Qureshi M, Salazar GA (2021). Pfam: The protein families database in 2021. Nucleic Acids Res.

[53] Song Y, DiMaio F, Wang RY-R, Kim D, Miles C (2013). High-resolution comparative modeling with RosettaCM. Structure.

[54] Sinha S, Lynn AM, Desai DK (2020). Implementation of homology based and non-homology based computational methods for the identification and annotation of orphan enzymes: using *Mycobacterium tuberculosis* H37Rv as a case study. BMC Bioinformatics.

[55] Botstein D, Fink GR (2011). Yeast: an experimental organism for 21st Century biology. Genetics.

[56] Brion C, Pflieger D, Souali-Crespo S, Friedrich A, Schacherer J (2016). Differences in Environmental Stress Response among Yeasts Is Consistent with Species-Specific Lifestyles.. Molecular Biology of the Cell.

[57] Hao Y, Zhang L, Niu Y, Cai T, Luo J (2018). SmProt: a database of small proteins encoded by annotated coding and non-coding RNA loci. Brief Bioinform.

[58] Hoang T, Nguyen DG, Bai WP, Oubridge X, Newman C (2016). Cryo-EM Structure of the Yeast U4/U6.U5 Tri-SnRNP at 3.7 Å Resolution. https://www.nature.com/articles/nature16940.

[59] Makarov EM, Makarova OV, Urlaub H, Gentzel M, Will CL (2002). Small nuclear ribonucleoprotein remodeling during catalytic activation of the spliceosome. Science.

[60] Hossain MA, Johnson TL (2014). Using yeast genetics to study splicing mechanisms. Methods Mol Biol.

[61] Vakirlis N, Hebert AS, Opulente DA, Achaz G, Hittinger CT (2018). A molecular portrait of *de novo* genes in yeasts. Mol Biol Evol.

[62] Andersson DI, Jerlström-Hultqvist J, Näsvall J (2015). Evolution of new functions *de novo* and from preexisting genes. Cold Spring Harb Perspect Biol.

[63] Li D, Dong Y, Jiang Y, Jiang H, Cai J (2010). A *de novo* originated gene depresses budding yeast mating pathway and is repressed by the protein encoded by its antisense strand. Cell Res.

