# Supplementary figures and images for: Identification of a novel gene required for competitive growth at high temperature in the thermotolerant yeast Kluyveromyces marxianus
Source: Microbiology (Reading). 2022 Mar 25;168(3):001148. doi: 10.1099/mic.0.001148 (PMC9558357; doi:10.1099/mic.0.001148)

Supplementary Figure 1.

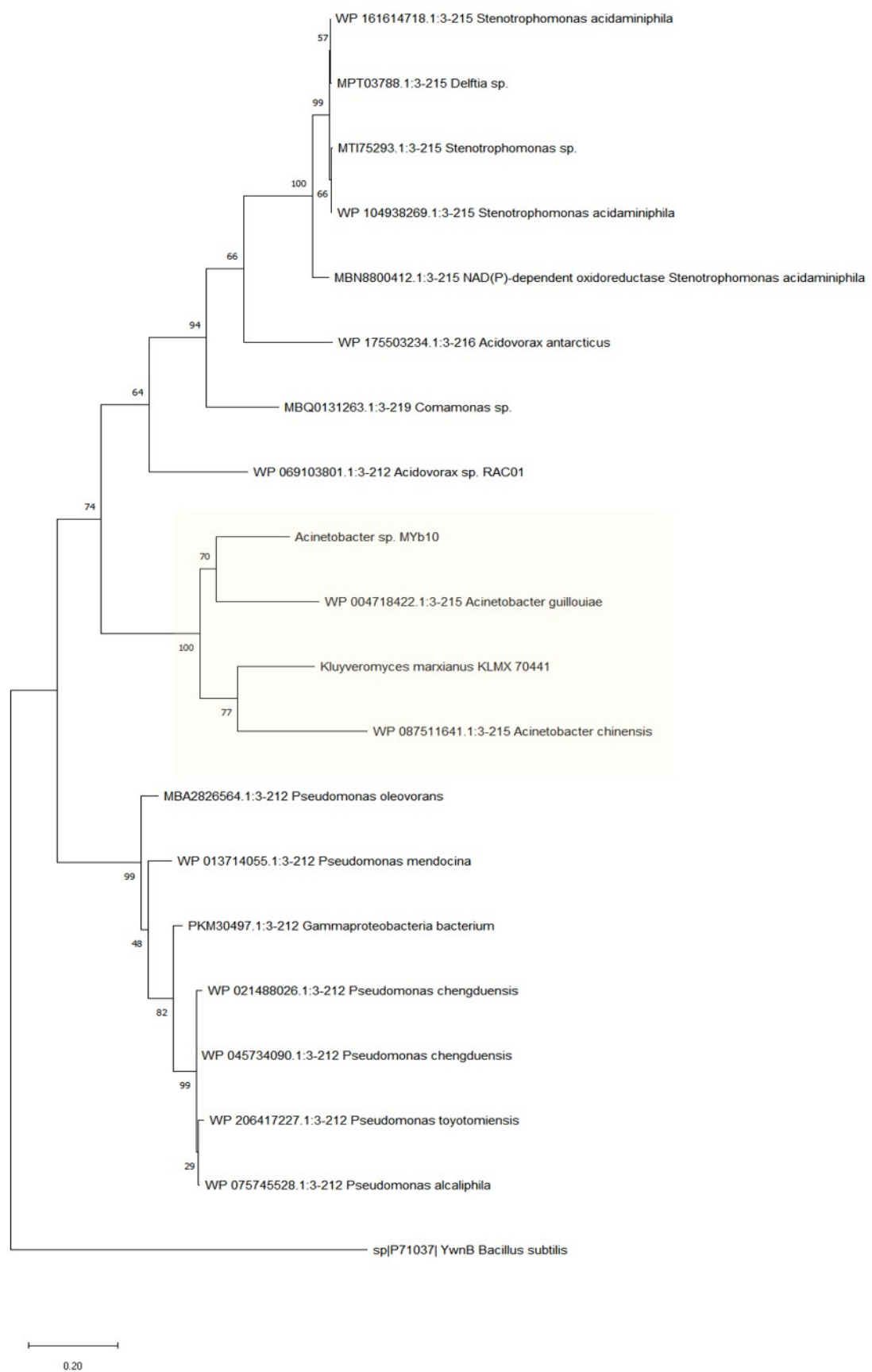

Supplementary Figure 2.

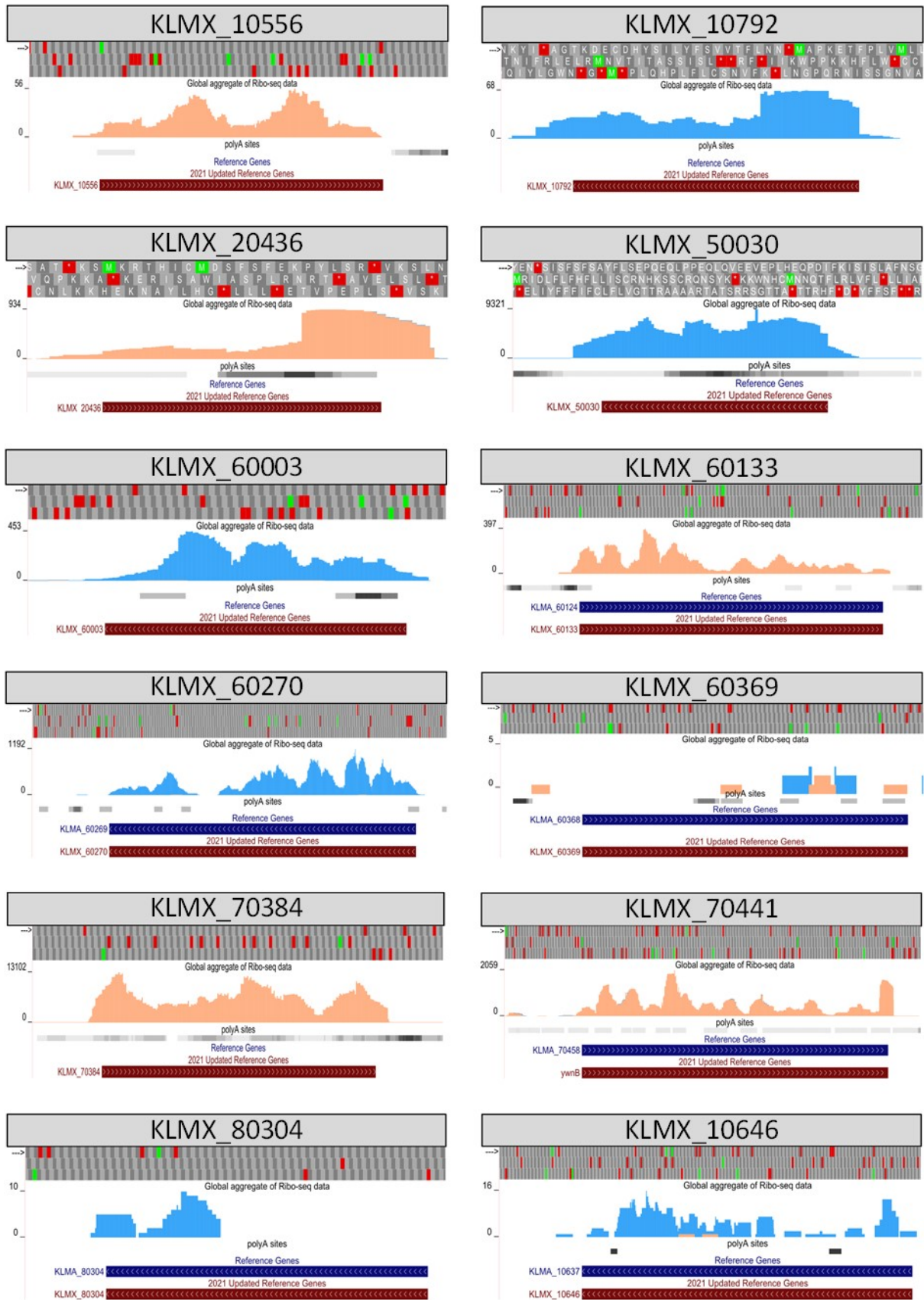

Supplementary Figure 3.

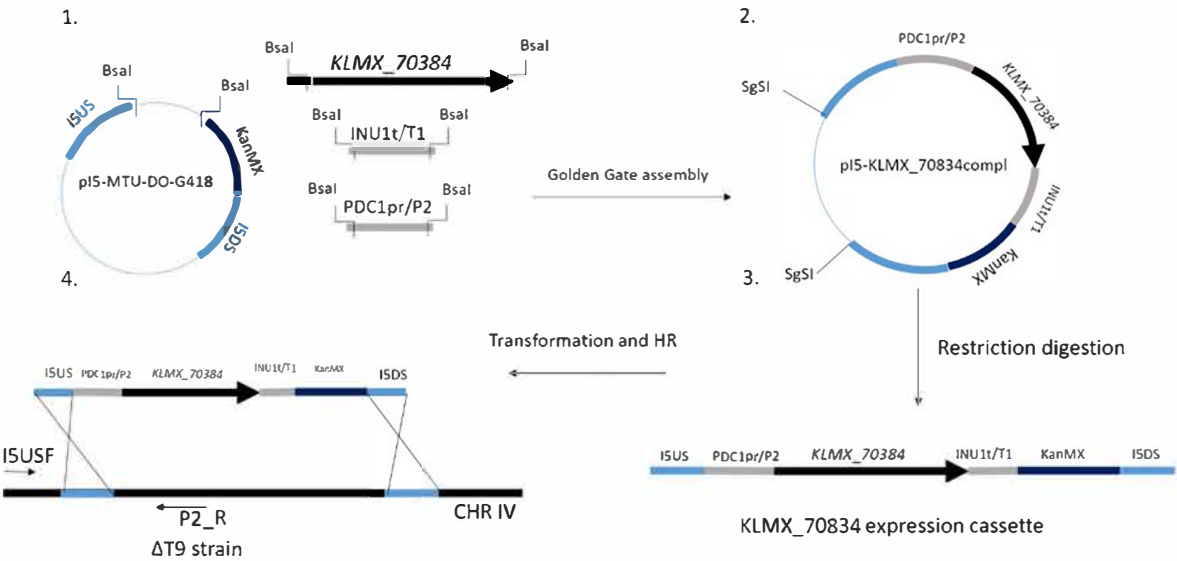

Supplement: Supplementary material 1 [file mic-168-1148-s001.pdf]
